# Unveiling Mesenchymal Stem Cells’ Regenerative Potential in Clinical Applications: Insights in miRNA and lncRNA Implications

**DOI:** 10.3390/cells12212559

**Published:** 2023-10-31

**Authors:** Maurycy Jankowski, Maryam Farzaneh, Farhoodeh Ghaedrahmati, Milad Shirvaliloo, Arash Moalemnia, Magdalena Kulus, Hanna Ziemak, Mikołaj Chwarzyński, Piotr Dzięgiel, Maciej Zabel, Hanna Piotrowska-Kempisty, Dorota Bukowska, Paweł Antosik, Paul Mozdziak, Bartosz Kempisty

**Affiliations:** 1 Department of Computer Science and Statistics, Poznan University of Medical Sciences, 60-812 Poznan, Poland; mjankowski@ump.edu.pl; 2 Department of Histology and Embryology, Poznan University of Medical Sciences, 60-781 Poznan, Poland; 3 Fertility, Infertility and Perinatology Research Center, Ahvaz Jundishapur University of Medical Sciences, Ahvaz, Iran; 4 Department of Immunology, School of Medicine, Isfahan University of Medical Sciences, Isfahan, Iran; 5 Infectious and Tropical Diseases Research Center, Tabriz University of Medical Sciences, Tabriz, Iran; 6 Future Science Group, Unitec House, 2 Albert Place, London N3 1QB, UK; 7 Faculty of Medicine, Dezful University of Medical Sciences, Dezful, Iran; 8 Department of Veterinary Surgery, Institute of Veterinary Medicine, Nicolaus Copernicus University in Torun, 87-100 Torun, Poland; 9 Division of Histology and Embryology, Department of Human Morphology and Embryology, Wroclaw Medical University, 50-368 Wroclaw, Poland; 10 Department of Physiotherapy, Wroclaw University School of Physical Education, 50-038 Wroclaw, Poland; 11 Division of Anatomy and Histology, University of Zielona Góra, 65-046 Zielona Góra, Poland; 12 Department of Toxicology, Poznan University of Medical Sciences, 60-631 Poznan, Poland; 13 Department of Basic and Preclinical Sciences, Institute of Veterinary Medicine, Nicolaus Copernicus University in Torun, 87-100 Torun, Poland; 14 Department of Diagnostics and Clinical Sciences, Institute of Veterinary Medicine, Nicolaus Copernicus University in Torun, 87-100 Torun, Poland; 15 Prestage Department of Poultry Science, North Carolina State University, Raleigh, NC 27607, USA; 16 Physiology Graduate Faculty, North Carolina State University, Raleigh, NC 27613, USA; 17 Division of Anatomy, Department of Human Morphology and Embryology, Wroclaw Medical University, 50-368 Wroclaw, Poland; 18 Department of Obstetrics and Gynecology, University Hospital and Masaryk University, 602 00 Brno, Czech Republic

**Keywords:** mesenchymal stem cells, miRNA, lncRNA

## Abstract

It is now widely recognized that mesenchymal stem cells (MSCs) possess the capacity to differentiate into a wide array of cell types. Numerous studies have identified the role of lncRNA in the regulation of MSC differentiation. It is important to elucidate the role and interplay of microRNAs (miRNAs) and long non-coding RNAs (lncRNAs) in the regulation of signalling pathways that govern MSC function. Furthermore, miRNAs and lncRNAs are important clinical for innovative strategies aimed at addressing a wide spectrum of existing and emerging disease. Hence it is important to consider their impact on MSC function and differentiation. Examining the data available in public databases, we have collected the literature containing the latest discoveries pertaining to human stem cells and their potential in both fundamental research and clinical applications. Furthermore, we have compiled completed clinical studies that revolve around the application of MSCs, shedding light on the opportunities presented by harnessing the regulatory potential of miRNAs and lncRNAs. This exploration of the therapeutic possibilities offered by miRNAs and lncRNAs within MSCs unveils exciting prospects for the development of precision therapies and personalized treatment approaches. Ultimately, these advancements promise to augment the efficacy of regenerative strategies and produce positive outcomes for patients. As research in this field continues to evolve, it is imperative to explore and exploit the vast potential of miRNAs and lncRNAs as therapeutic agents. The findings provide a solid basis for ongoing investigations, fuelling the quest to fully unlock the regenerative potential of MSCs.

## 1. Introduction

Mesenchymal stem cells (MSCs) are multipotent cells that can differentiate into a variety of cell types, including bone, cartilage, muscle, and fat cells. They are commonly isolated from bone marrow but can also be found in other tissues, such as adipose tissue and the umbilical cord. MSCs are attractive for medical applications due to their ability to migrate to sites of injury or inflammation and their potential to differentiate into cells that can repair damaged tissue [1]. In addition, MSCs have immunomodulatory properties, making them useful for treating conditions such as autoimmune disorders and graft-versus-host disease. MSCs can be expanded in culture and manipulated ex vivo to promote specific cellular differentiation and are considered a promising tool for regenerative medicine [2]. However, further research is needed to fully understand the mechanisms underlying MSC function and to optimize their use for various clinical applications.

MicroRNAs (miRNAs) are small, non-coding RNA molecules that play a critical role in the regulation of gene expression. miRNAs bind target messenger RNA (mRNA) molecules, leading to their degradation or inhibition, preventing them from being translated into proteins [3]. This allows miRNAs to regulate the expression of multiple genes, making them an important component of gene regulation and cellular function. miRNAs have been shown to play a key role in regulating gene expression, and to be involved in a wide range of biological processes, including development, cell growth and division, and apoptosis [4,5,6,7]. miRNAs have also been implicated in the development and progression of various diseases, including cancer, cardiovascular disease, and neurological disorders [8,9,10]. By regulating the expression of genes involved in disease, miRNAs can act as either oncogenes or tumour suppressors [11,12,13,14].

The involvement of miRNA in a multitude of diseases makes them potential biomarkers for diagnostics as well as therapeutic tools, targeting genes responsible for a specific condition [15,16,17]

Furthermore, miRNAs play a crucial role in regulating MSC differentiation into various cell types, such as bone and cartilage [18,19]. MSCs can secrete miRNAs that promote or inhibit the differentiation of neighbouring cells [20]. The regulation of miRNAs in MSC differentiation is complex, and the role of specific miRNAs in the process is still being elucidated.

miRNAs exert a crucial influence on the intricate regulation of MSCs. Notably, certain miRNAs have been identified as key regulators of the immunosuppressive properties possessed by MSCs, underscoring their significance in unlocking the full therapeutic potential of these cells [21,22]. By introducing specific miRNAs into MSCs, researchers can target and tailor their therapeutic effects for specific diseases or conditions [23,24]. For instance, engineering MSCs to express anti-inflammatory miRNAs holds promise for combating inflammatory diseases, while harnessing miRNAs that promote tissue repair could revolutionize the treatment of tissue injuries [25,26]. This intersection of miRNAs and MSC engineering offers a promising frontier for advancing regenerative medicine and personalized therapeutic interventions.

Long non-coding RNAs (lncRNAs) are RNA molecules that are longer than 200 nucleotides but do not encode proteins [27,28]. Unlike protein-coding mRNA, lncRNA do not have a conserved open reading frame and are not translated into proteins. Despite their lack of coding capacity, lncRNA play critical roles in gene regulation and cellular processes. They have been shown to act as epigenetic regulators, scaffolds for protein complexes, and decoys for miRNA, among other functions [29,30,31,32,33]. lncRNA can also serve as molecular markers for various diseases, including cancer, and can be used for diagnostic and prognostic purposes [34,35,36]. The discovery of lncRNA has expanded our understanding of the diversity and complexity of RNA-mediated gene regulation and has opened up new avenues for the development of therapeutic strategies [37]. However, much remains to be learned about the full extent of lncRNA functions and the mechanisms underlying their effects on gene expression [38,39].

A number of studies have identified lncRNA as playing a key role in regulating MSC differentiation into various cell types [40,41]. For example, the lncRNA HOTAIR has been shown to regulate the differentiation of MSCs into osteoblasts [42]. In addition, the lncRNA MALAT1 has been shown to promote the ability of MSCs to form new blood vessels and promote proliferation [43]. Studies have highlighted the potential application of lncRNAs as innovative biomarkers for diagnosis and as potential targets for therapeutic treatments [44,45,46].

## 2. Characteristics and Function of MSCs

**Figure 1:**
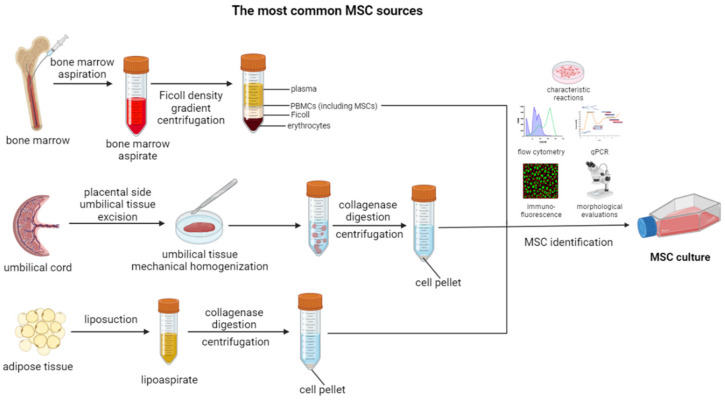
Overview of the most common MSC sources and methods of their isolation. Created with Biorender.com.

MSCs are a type of stem cell that have the ability to differentiate into a variety of cell lines, including bone, cartilage, muscle, and fat cells. They are commonly isolated from bone marrow, but they can also be found in other tissues, such as adipose tissue and umbilical cord (Figure 1) [47,48,49]. MSCs exhibit a range of characteristic properties, which enable their identification, as well as facilitate the range of their physiological functions (Figure 1) [50].

**Figure 2:**
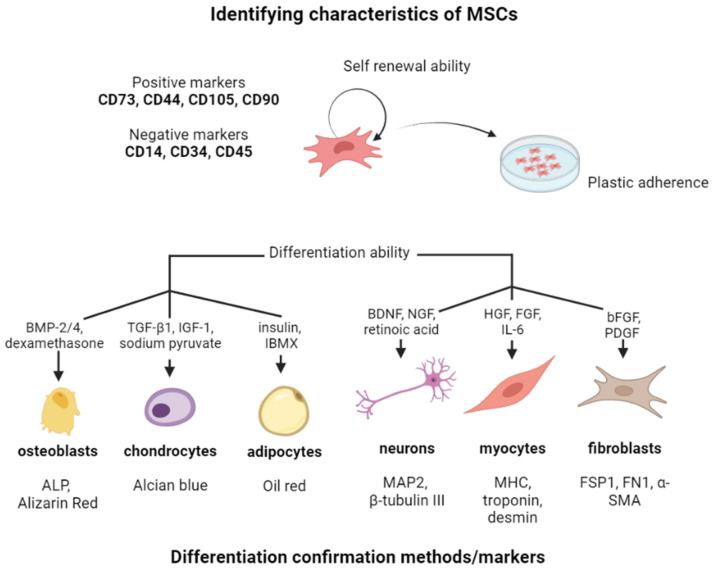
The overview of the identifying characteristics of MSCs. Created with Biorender.com.

MSCs are characterized by specific cell surface markers such as CD73, CD90, and CD105, and lack the expression of hematopoietic cell markers like CD45, CD34, and CD14. These markers are used to identify and isolate MSCs from other cell types [51]. Moreover, there is a number of characteristic properties, that further allow to identify MSCs among other stem cell populations (Figure 2).

MSCs are characterized by their multipotency, which means that they have the ability to differentiate into multiple cell types, including osteocytes, chondrocytes, adipocytes, and myocytes [52,53]. MDC differentiation potential makes them an important tool for regenerative medicine and tissue engineering [2]. The process of MSC differentiation is regulated by a variety of factors, including growth factors, cytokines, and the extracellular matrix. Differentiation involves a series of molecular events that result in changes in gene expression and cell morphology. MSC differentiation can be induced by specific factors, such as dexamethasone, ascorbic acid, and beta-glycerophosphate for osteogenic differentiation, transforming growth factor-beta (TGF-beta) and bone morphogenetic protein-2 (BMP-2) for chondrogenic differentiation, and insulin and dexamethasone for adipogenic differentiation [53,54]. Osteogenic differentiation is the process by which MSCs differentiate into osteoblasts, which are cells responsible for bone formation. During osteogenic differentiation, MSCs undergo changes in gene expression and cell morphology that result in the production of bone matrix proteins, such as collagen and osteocalcin. The resulting osteoblasts then mineralize the bone matrix to form new bone tissue [55,56]. Chondrogenic differentiation is the process where MSCs differentiate into chondrocytes, which are cells responsible for cartilage formation. During chondrogenic differentiation, MSCs undergo changes in gene expression and cell morphology that result in the production of cartilage matrix proteins, such as collagen and aggrecan [57,58]. The resulting chondrocytes then produce a cartilage matrix that can be used for tissue engineering applications [58]. Adipogenic differentiation is the process in which MSCs differentiate into adipocytes, which are cells responsible for fat storage [59]. During adipogenic differentiation, MSCs undergo changes in gene expression and cell morphology that result in the production of lipid droplets. The resulting adipocytes can be used for tissue engineering applications, such as the development of adipose tissue for reconstructive surgery [60,61]. Finally, myogenic differentiation is the process where MSCs differentiate into myocytes, which are cells responsible for muscle formation [62,63]. During myogenic differentiation, MSCs undergo changes in gene expression and cell morphology that result in the production of myogenic proteins, such as MyoD and myogenin. The resulting myocytes can be used for tissue engineering applications, such as the development of muscle tissue for reconstructive surgery [64].

Furthermore, MSCs have the ability to self-renew, which means that they can freely proliferate to create the exact copies of themselves in an almost indefinite manner. This ability is essential for the maintenance of a pool of MSCs in the body that can be used for tissue regeneration and repair when needed. Self-renewal is a complex process that involves several mechanisms. One of the key factors involved in self-renewal is the expression of specific genes that regulate stem cell function. In MSCs, the expression of genes such as Sox2, Oct4, and Nanog has been found to be important for self-renewal [65,66]. The process of self-renewal is strongly influenced by growth factors and cytokines, as they play a crucial role in signaling mesenchymal stem cells (MSCs) to retain their stem cell characteristics and undergo division, resulting in the generation of additional stem cells [67,68,69,70]. For example, fibroblast growth factor-2 (FGF-2) is important for the self-renewal of MSCs [69,71]. The extracellular matrix (ECM) is a complex network of proteins and other molecules that surrounds cells and provides structural support which plays an important role in MSC self-renewal [72]. Interactions with the ECM modulate MSCs’ behaviour, including their self-renewal capacity. Notably, a laminin peptide, an ECM molecule, has been identified as a promoter of MSC self-renewal [73]. Finally, the microenvironment, or niche, where MSCs reside, plays a crucial role in their self-renewal. Within this niche, MSCs receive specific signals that govern their behaviour, including the capacity to self-renew. For instance, The hypoxic microenvironment is crucial for maintaining undifferentiated MSCs by keeping them quiescent and promoting necessary self-renewal. Hypoxia inducible factor (HIF) acts as a molecular regulator within this environment, controlling MSC differentiation and survival [74].

Moreover, MSCs have immunomodulatory properties, they can regulate the various elements of the immune system. They can suppress the activity of T-cells and other immune cells, reducing inflammation and preventing immune-mediated tissue damage [75]. MSCs can aid in tissue repair and regeneration by secreting factors that promote the growth and activity of immune cells and anti-inflammatory factors that can reduce inflammation and promote tissue repair [76]. These factors include interleukin-10 (IL-10), transforming growth factor-beta (TGF-β), and prostaglandin E2 (PGE2) [77,78]. MSCs can also secrete factors that promote the growth of new blood vessels, a process known as angiogenesis. This function can play an important role in in repairing damaged tissues that require a new source of blood supply [79,80]. Furthermore, MSCs have been shown to have neuroprotective properties, meaning they can protect neurons from damage and promote their survival [81]. They can secrete factors that promote nerve cell growth and regeneration, making them a potential therapy for neurological disorders [82]. MSCs can also promote wound healing by secreting growth factors that promote the growth of new skin cells and blood vessels [83,84]. Finally, MSCs are able to remodel the extracellular matrix (ECM) of tissues. The ECM is the complex network of proteins and other molecules that provides structural support to tissues. MSCs can produce enzymes that break down and remodel the ECM, which is important for tissue repair and regeneration [85].

In conclusion, MSCs have a wide range of known physiological functions in the body, including tissue repair and regeneration, immune modulation, anti-inflammatory effects, angiogenesis, and neuroprotection. It also needs to be noted that these cells are a subject of continuous research, indicating that there might by a wide array of yet undiscovered functions that could bring additional promise to their application in further fields of science and medicine.

## 3. Preclinical Studies, Clinical Trials, and Therapies

Preclinical studies, clinical trials, and therapies involving mesenchymal stem cells (MSCs) are aimed at exploring the therapeutic potential of these cells in various diseases and conditions. The development of MSC-based therapies has been driven by their unique characteristics, including the ability to self-renew, differentiate into various cell types, and exert immunosuppressive effects [86]. Preclinical studies are conducted in laboratory settings or in animals, and are used to evaluate the safety and efficacy of MSCs before they can be tested in humans. These studies have demonstrated that MSCs have the potential to regenerate damaged tissues, reduce inflammation, and promote tissue repair [1]. MSCs have been shown to improve outcomes in preclinical models of a range of diseases and conditions, including heart disease, osteoarthritis, liver disease, and spinal cord injury, among [87,88,89]. MSC-based therapies involve the administration of MSCs directly to patients with the aim of treating specific diseases or conditions. MSCs can be delivered to patients either through injections into the affected tissues or intravenously. MSCs are capable of homing to damaged tissues and promoting tissue repair through mechanisms such as secreting growth factors, reducing inflammation, and inducing angiogenesis [53,79]. Clinical trials are conducted in humans to evaluate the safety and efficacy of MSC-based therapies. Clinical trials involving MSCs are currently underway in various stages, ranging from phase I to phase III. Phase I trials are usually small and focus on evaluating the safety of MSC treatments, while phase II and III trials are larger and focus on evaluating the efficacy of MSC treatments. The results of these trials have been promising, with MSCs showing the potential to treat a range of diseases and conditions, including osteoarthritis, Crohn’s disease, heart failure, and spinal cord injury. The currently completed and terminated studies related to MSCs were presented in Table 1. Furthermore, according to the ClinicalTrials.gov database, there are 318 ongoing clinical trials related to mesenchymal stem cells, in different completion stages, with no results yet reported.

While the potential for MSCs in regenerative medicine is vast, there are still many challenges that need to be overcome. One of the major challenges is to ensure the safety and efficacy of MSC treatments, which requires rigorous preclinical and clinical testing [90]. Additionally, the high cost of MSC treatments, as well as the limited availability of funding and insurance coverage, continue to be major barriers to their widespread use.

In conclusion, preclinical studies, clinical trials, and MSC-based therapies are contributing to the development of new treatments for a range of diseases and conditions. While the results of these studies have been promising, further research is needed to fully understand the mechanisms of action of MSCs and to determine their safety and efficacy in the treatment of specific diseases and conditions [90]. Nevertheless, MSCs hold great promise as a new class of regenerative therapies, and their continued development and testing is essential to realizing their full therapeutic potential.

## 4. MSC Differentiation

Based on their ability to differentiate, MSCs support tissue homeostasis by acting as a source of renewable progenitor cells for the repair of damaged tissues and the replacement of cells in routine cellular turnover throughout adult life [91,92,93]. When cultured under specific conditions, they can differentiate into multiple mesenchymal lineage cell types, including osteoblasts, chondrocytes, adipocytes, and myoblasts [94,95,96,97]. The classical method for osteogenic differentiation of human MSCs involves incubation in fetal bovine serum (FBS)-containing medium supplemented with ascorbic acid, β-glycerophosphate, and dexamethasone, resulting in an increase in calcium accumulation and alkaline phosphatase activity [98,99]. Chondrogenic differentiation is accomplished using pelleted micromass cultured in the presence of transforming growth factor (TGF)-β in serum-free medium, which produces cartilage-specific, highly sulfated proteoglycans and type II collagen [98]. Adipogenic differentiation of MSCs is demonstrated through the detection of lipid vacuoles after dexamethasone, insulin, isobutyl methyl xanthine, and indomethacin are added to medium containing FBS [9]. MSCs can also differentiate into myoblasts when treated with 5-azacytidine and amphotericin B, which fuse into rhythmically beating myotubes [100]. Furthermore, MSCs can also give rise to cross-lineage cell types such as endodermal-hepatocytes and β-cells of pancreatic islets and ectodermal-neurons, a process known as trans-differentiation [101,102]. The liver cells were obtained from MSCs in two stages by culturing them in Iscove’s modified Dulbecco’s medium (IMDM) supplemented with HGF, bFGF and nicotinamide, and in the next stage with the addition of oncostatin M, dexamethasone, and ITS+ (insulin, transferring, selenium). Albumin, α-fetoprotein, and hepatocyte nuclear factor 4 (HNF-4) are present in the resulting cells, which are hepatocyte typical markers [103]. Pancreatic islets of β-cells capable of producing insulin were obtained from MSCs by treating them with a mixture of growth factors secreted by regenerating cells of the pancreas and also by using acitin A, sodium butyrate, taurine, and nicotinamide [104,105]. According to Hofstetter and colleagues, neuron-like cells differentiated from MSCs lack voltage-gated ion channels that are required for action potential generation; thus, they may not be considered as true neurons [106]. Additionally, transdifferentiate of MSCs into endothelial cells expressing endothelial nitric oxide synthase have been reported that contribute to endothelial function improvement in vascular injury rat model [107,108]. There has been widespread evidence that miRNAs and lncRNAs play an important role in the differentiation of MSCs, both positively and negatively, as reported herein (Table 2 and Table 3).

## 5. Signalling Pathways Governing MSC Function

**Figure 3:**
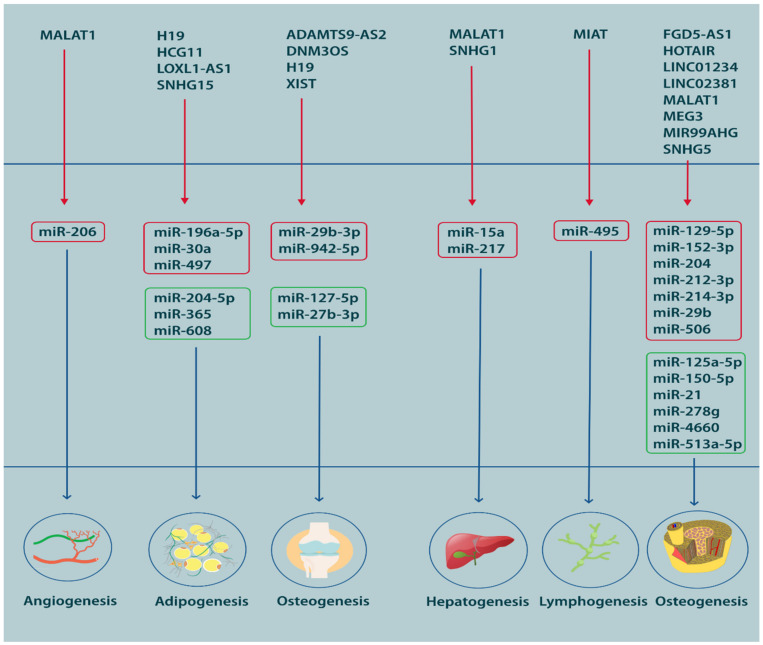
A visual representation of miRNA participation in the process of MSC differentiation.

Based on the widely accepted definition of ‘tissue engineering’ that was proposed by Robert Nerem in 1988, MSCs can be regarded as an inherent component of the modern regenerative medicine, since they can readily be used for the generation of different cell lineages. The growing success of today’s regenerative medicine stems from the pluripotent nature of MSCs that renders them capable of transforming into other cell types with regards to their microenvironment, which consists of non-coding RNAs, among others [303]. A strikingly high proportion of studies have focused on identification of ncRNAs that facilitate or impair the differentiation of MSCs. These ncRNAs usually constitute an elaborate network or axis of interactions involving lncRNAs, miRNAs, mRNAs and other types of ncRNAs, which can ultimately affect the proliferative and regenerative activity of these cells. Generally, in RNA-based regulatory pathways, lncRNAs bind and sponge miRNAs to indirectly promote the translation of certain mRNAs to their final product. As such, a basic lncRNA/miRNA/mRNA pathway includes an inhibitory pathway accompanied by an indirect de-repressing effect. While a range of other lncRNAs and miRNAs might be involved in this inhibitory process, they usually are the final effector molecule that determines the final cell fate [304]. For instance, if the axis ends in ‘vascular endothelial growth factor’ (VEGF) with a net de-repressing or stimulatory effect, the MSCs occurring in that microenvironment will be compelled to differentiate into endothelial cells, giving rise to vasculature [305]. In addition to microenvironmental properties, the biological origin of MSCs may influence the course of differentiation. Bone marrow, umbilical cord, adipose tissue, peripheral blood and synovium stand among the most frequently preferred sources of MSCs in experimental and clinical applications. Despite being pluripotent, MSCs are still subject to epigenetic regulatory programs associated with the source from which they are derived. In this sense, MSCs extracted from the synovial space are theoretically anticipated to yield better results when used for cartilage regeneration in joint disorders [306]. Still, there are no strict rules regarding the source, as there are reports of successful trials of seemingly contrasting sources for regenerative purposes such as application of adipose-derived MSCs for osteogenic regeneration in patients with osteoarthritis [307], suggesting, once again, that environmental factors and regulatory pathways are as important as the source. Figure 3 illustrates various miRNAs that are critical during MSCs differentiation.

The lnRNA-miRNA basis of MSC differentiation has primarily been studied in the case of osteogenic, chondrogenic and adipogenic differentiation. More scantly, the role of ncRNAs in hepatogenic, angiogenic and lymphatogenic differentiation has also been explored, albeit, to a much lesser extent. Induction of osteogenic differentiation is of utmost importance in the treatment of degenerative bone diseases. Accordingly, regulatory lncRNAs and miRNAs can be used as therapeutic agents or targets with regards to their stimulatory or inhibitory effects, respectively. One good example is ‘metastasis associated lung adenocarcinoma transcript 1′ (MALAT1), a tumor-associated lncRNA with known osteogenic effects [308,309]. Considering its mechanism of action, MALAT1 can either be used as an exogenous therapeutic agent for induction of osteogenesis or targeted by proxy when it is lowly expressed. Downregulation of MALAT1, as an osteogenic lncRNA, results in de-repression of anti-osteogenic miRNAs, which can be targeted and silenced using specialized short hairpin RNAs (shRNAs) [308,309]. Though, differentiation is not necessarily a desirable outcome, particularly when it comes to malignancies. MALAT1, which is a beneficial factor in the case of hypoproliferative disorders, may assume an adverse role in the context of oncogenesis, where overexpression of MALAT1 stimulates formation of new endothelial cells, hence, promoting angiogenesis in osteosarcoma [310]. However, the regulation of differentiation is important in treating disorders associated with impaired formation or degeneration of vasculature, which may benefit from overexpression of MALAT1 [305]. One reason for this presumable divergent function of MALAT1, or any other lncRNA for that matter, is the difference in miRNAs which are targeted and sponged in each scenario. When it is a beneficial pro-angiogenic factor, MALAT1 targets miR-206 to upregulate VEGFA in the population of endothelial cells that might be overproducing the anti-angiogenic miR-206 [305]. When it is an aggravator of tumor-associated angiogenesis, MALAT1 targets the anti-angiogenic miR-150-5p, when it should not be sponged [310]. In this sense, a good understanding of the lncRNA-miRNA networks governing cell differentiation in health and disease can substantially contribute to the performance of regenerative medicine. The full overview of knowledge regarding the participation of miRNAs and lncRNAs in differentiation of MSCs, as well as the different lncRNA-miRNA axes regulates differentiation into different lineages (Table 4).

## 6. Practical Implications and Future Perspective of lncRNA and miRNA in MSCs Treatment

Numerous studies highlight the potential of mesenchymal stem cells (MSCs) in repairing various organs like the lungs, heart, and skin. Exosomes, tiny vesicles produced by MSCs, have gained importance in regenerative medicine [335]. Exosomes, packed with RNA and proteins, are safer and more stable than direct MSC transplants [336]. They play a crucial role in healing by delivering therapeutic substances, especially microRNAs (miRNAs), which regulate gene activity in nearby or distant cells [337].Studies show that MSC-derived exosomes can transport miRNAs, such as miR-132–3p, to endothelial cells, improving their growth and reducing blood-brain barrier dysfunction in a brain injury model [338]. These exosomes boost the expression of essential genes in traumatic brain injury.

Exosomes and miRNAs offer promise in treating various diseases, including neurological, cardiovascular, and kidney disorders. Exosomes containing specific miRNAs have beneficial effects on neurological conditions, reducing cell death and inflammation. MiRNAs like miR-126 and miR-184 help brain recovery in stroke models [339]. In autoimmune encephalomyelitis, BM-MSC exosomes deliver miR-367–3p, reducing symptoms [340]. MSC-derived exosomes are also promising in cardiovascular diseases. They target specific genes, reducing inflammation and improving heart function. For example, exosomes containing miR-149 have been used to target genes and modulate the inflammatory response [341]. In kidney repair, they counter calcification and promote recovery. Exosomes containing miR-874-3p have been shown to control necroptosis, decrease renal tubular cell damage, and improve healing in acute kidney injury [342]. For liver issues, exosomes enriched with miR-148a mitigate symptoms, and miR-20a-5p promotes liver repair [343,344]. Lung diseases, arthritis, and osteoarthritis also show potential for exosome therapy. Lung diseases, such as cystic fibrosis, pulmonary fibrosis, and radiation-induced lung injury, have been studied in the perspective of exosomal therapy. MiR-466f-3p and miR-186 have shown therapeutic potential in reducing inflammation, fibrosis, and promoting repair [345,346]. In the case of rheumatoid arthritis, exosomes containing miR-150-5p have been used to downregulate MMP14 and VEGF, reducing inflammation and protecting against cartilage and bone degradation [347]. Exosomes can be used to encourage direct intracellular transfer of miRNAs between cells, thereby promoting anti-inflammatory effects. Osteoarthritis has been studied in the context of BMP2-induced chondrogenesis and the Wnt signaling pathway. Exosomal miR-181c-5p and miR-92a-3p have been implicated in cartilage repair and Wnt inhibition [348,349].

LncRNAs have shown exciting potential in addressing various health conditions and guiding MSCs through various cellular processes. In osteogenic differentiation, LncRNAs like H19, HULC, and MALAT1 exert their influence, promoting bone formation through mechanisms involving miRNAs and key signaling pathways [279,286,287]. Notably, researchers have uncovered a distinctive LncRNA, lncRNA-OG, driving bone growth alongside hnRNPK, which could pave the way for better bone-related treatments [289]. While the immunoregulatory potential of MSCs is significant, only a few studies, like one involving LncRNA-MALAT1, have delved into this arena [43]. Investigating LncRNA-driven immune regulation in MSCs is an area rich in potential. Furthermore, LncRNAs including Lnc-ZNF354A, Lnc-LIN54, Lnc-FRG2C, and Lnc-USP50, were found to be closely associated with pathological bone formation in ankylosing spondylitis [350].Adipogenic differentiation, the process of forming fat cells, is also influenced by LncRNAs such as GAS5 and HOTAIR [299,300]. The balance between osteogenesis and adipogenesis in MSCs is delicately controlled by LncRNAs like H19 and TCONS_00041960, offering a potential therapeutic angle for conditions like osteoporosis [292,296]. Interestingly, LncRNA lnc13728 surfaces, significantly influencing the proliferation of fat cells and modulating genes associated with obesity, presenting opportunities to tackle obesity-related challenges more effectively [298]. In the context of chondrogenic differentiation, LncRNAs like ZBED3-AS1 steer MSCs toward the formation of cartilage tissue, influencing pivotal pathways such as Wnt/β-catenin and offering prospects for therapeutic interventions, particularly in conditions like osteoarthritis [295]. Venturing into the realms of neurogenesis, myogenesis, and endothelial differentiation, LncRNAs like H19, MIAT, MEG3, and HULC actively contribute to the formation of neural, smooth muscle, and endothelial cells [351,352]. Their roles in addressing nerve injuries and cardiovascular therapies beckon for deeper exploration.

Meanwhile, the impact of exosomes from lung cancer on the LncRNA expression profile of MSCs emphasizes the participation of LncRNAs in the intricate interplay between MSCs and tumor cells, ultimately affecting the progression of diseases [353]. This underscores the potential of using LncRNA profiles in circulating MSCs as personalized diagnostic tools for specific medical conditions. Circulating MSCs in peripheral blood hold promise as diagnostic markers for various diseases, offering a novel and precise diagnostic method by identifying specific LncRNAs or patterns within these MSCs. Furthermore, there is an uncharted frontier in enhancing the clinical effectiveness of MSC-based therapies by manipulating LncRNAs that govern MSC behavior. Employing gene editing techniques to fine-tune specific LncRNA expressions has the potential to enhance the immunoregulatory capabilities of MSCs in autoimmune diseases and guide their differentiation into specialized cell types for tissue and regeneration engineering. This dynamic approach opens exciting avenues for refining MSC-based therapies across various diseases.

Overall, LncRNAs are master conductors of MSC behavior, orchestrating a symphony of cellular functions, from differentiation to proliferation and immunoregulation. while, exosomes and miRNAs have opened exciting avenues in regenerative medicine, offering hope for various health conditions. Understanding and harnessing the power of LncRNAs in MSCs offer promising avenues for innovative therapeutics and regenerative medicine.

## 7. Conclusions

It is important to consider the intricate regulatory roles of miRNAs and lncRNAs in governing the signalling pathways that dictate MSC functioning and differentiation. The findings presented underscore the pivotal significance of these small RNA molecules in the realm of regenerative medicine and hold great promise for future therapeutic applications. The characterization and functional attributes of MSCs have been thoroughly examined, revealing their remarkable potential in tissue repair and immune modulation. As highlighted by an array of preclinical studies, clinical trials, and innovative therapies, MSCs have demonstrated their transformative capability in addressing diverse medical conditions, further emphasizing their significance as a regenerative resource. The emerging understanding of lncRNAs as key modulators of lineage commitment. The intricate interplay between lncRNAs and signalling pathways provides crucial insights into the mechanisms governing MSC fate determination, offering opportunities for targeted interventions and precision therapeutics. Furthermore, the regulatory impact of miRNAs on MSC differentiation has been comprehensively analysed, unravelling the complexity of gene expression network. The interplay between miRNAs and their target genes offers a deep understanding of the regulatory landscape driving MSC differentiation processes, paving the way for potential therapeutic strategies targeting these molecular interactions.

In conclusion, the knowledge amassed serves as a crucial foundation for further advancements in regenerative medicine. Harnessing the regulatory potential of miRNAs and lncRNAs in MSCs presents exciting prospects for developing targeted therapies and personalized treatment approaches, ultimately enhancing the efficacy of regenerative strategies and positively impacting patient outcomes. As research in this field continues to evolve, it is imperative to explore and exploit the vast potential of miRNAs and lncRNAs as therapeutic agents. The findings presented here provide a solid basis for ongoing investigations, fuelling the quest to fully unlock the regenerative potential of MSCs.

## Figures and Tables

**Table 1:** Compilation of completed and terminated studies related to the use of MSCs, obtained from the ClinicalTrials.gov database.

Nr	NCT Number	Title	Status	Conditions	Phases	URL, (All Accessed 5 September 2023)
1	NCT02866721	Safety and Tolerability Study of Allogeneic Mesenchymal Stem Cell Infusion in Adults With Cystic Fibrosis	Completed	Cystic Fibrosis	Phase 1	https://ClinicalTrials.gov/show/NCT02866721
2	NCT01775774	Human Mesenchymal Stem Cells For Acute Respiratory Distress Syndrome	Completed	Acute Respiratory Distress Syndrome	Phase 1	https://ClinicalTrials.gov/show/NCT01775774
3	NCT02387749	Effect Of Mesenchymal Stem Cells Transfusion on the Diabetic Peripheral Neuropathy Patients.	Completed	Diabetic Peripheral Neuropathy	Not Applicable	https://ClinicalTrials.gov/show/NCT02387749
4	NCT01932164	Use of Mesenchymal Stem Cells for Alveolar Bone Tissue Engineering for Cleft Lip and Palate Patients	Completed	Cleft Lip and Palate	Not Applicable	https://ClinicalTrials.gov/show/NCT01932164
5	NCT02481440	Repeated Subarachnoid Administrations of hUC-MSCs in Treating SCI	Completed	Spinal Cord Injuries	Phase 1|Phase 2	https://ClinicalTrials.gov/show/NCT02481440
6	NCT01856140	Treatment of Tendon Injury Using Mesenchymal Stem Cells	Completed	Lateral Epicondylitis	Early Phase 1	https://ClinicalTrials.gov/show/NCT01856140
7	NCT02330978	Intravitreal Mesenchymal Stem Cell Transplantation in Advanced Glaucoma.	Completed	Retinal Degeneration|Primary Open-angle Glaucoma	Phase 1	https://ClinicalTrials.gov/show/NCT02330978
8	NCT01183728	Treatment of Knee Osteoarthritis With Autologous Mesenchymal Stem Cells	Completed	Osteoarthritis, Knee|Knee Degenerative Disease|Knee Osteoarthritis	Phase 1|Phase 2	https://ClinicalTrials.gov/show/NCT01183728
9	NCT01586312	Treatment of Knee Osteoarthritis With Allogenic Mesenchymal Stem Cells	Completed	Osteoarthritis, Knee|Arthritis of Knee|Knee Osteoarthritis	Phase 1|Phase 2	https://ClinicalTrials.gov/show/NCT01586312
10	NCT02298023	Treatment of Tendon Injury Using Allogenic Adipose-derived Mesenchymal Stem Cells (Rotator Cuff Tear)	Completed	Rotator Cuff Tear	Phase 2	https://ClinicalTrials.gov/show/NCT02298023
11	NCT00587990	Prospective Randomized Study of Mesenchymal Stem Cell Therapy in Patients Undergoing Cardiac Surgery (PROMETHEUS)	Terminated	Stem Cell Transplantation|Ventricular Dysfunction, Left	Phase 1|Phase 2	https://ClinicalTrials.gov/show/NCT00587990
12	NCT03102879	Encapsulated Mesenchymal Stem Cells for Dental Pulp Regeneration.	Completed	Periapical Periodontitis	Not Applicable	https://ClinicalTrials.gov/show/NCT03102879
13	NCT02065245	AllogeneiC Human Mesenchymal Stem Cells (hMSC) in Patients With Aging FRAilTy Via IntravenoUS Delivery	Completed	Frailty	Phase 1|Phase 2	https://ClinicalTrials.gov/show/NCT02065245
14	NCT04313647	A Tolerance Clinical Study on Aerosol Inhalation of Mesenchymal Stem Cells Exosomes In Healthy Volunteers	Completed	Healthy	Phase 1	https://ClinicalTrials.gov/show/NCT04313647
15	NCT01385644	A Study to Evaluate the Potential Role of Mesenchymal Stem Cells in the Treatment of Idiopathic Pulmonary Fibrosis	Completed	Idiopathic Pulmonary Fibrosis	Phase 1	https://ClinicalTrials.gov/show/NCT01385644
16	NCT02513238	Mesenchymal Stemcells for Radiation Induced Xerostomia	Completed	Xerostomia	Phase 2	https://ClinicalTrials.gov/show/NCT02513238
17	NCT02501811	Combination of Mesenchymal and C-kit+ Cardiac Stem Cells as Regenerative Therapy for Heart Failure	Completed	Ischemic Cardiomyopathy	Phase 2	https://ClinicalTrials.gov/show/NCT02501811
18	NCT02509156	Stem Cell Injection in Cancer Survivors	Completed	Cardiomyopathy Due to Anthracyclines	Phase 1	https://ClinicalTrials.gov/show/NCT02509156
19	NCT02379442	Early Treatment of Acute Graft Versus Host Disease With Bone Marrow-Derived Mesenchymal Stem Cells and Corticosteroids	Terminated	Graft-Versus-Host Disease	Phase 1|Phase 2	https://ClinicalTrials.gov/show/NCT02379442
20	NCT02013674	The TRansendocardial Stem Cell Injection Delivery Effects on Neomyogenesis STudy (The TRIDENT Study)	Completed	Chronic Ischemic Left Ventricular Dysfunction|Myocardial Infarction	Phase 2	https://ClinicalTrials.gov/show/NCT02013674
21	NCT03691909	Phase 1/2a Clinical Trial to Assess the Safety of HB-adMSCs for the Treatment of Rheumatoid Arthritis	Completed	Rheumatoid Arthritis	Phase 1|Phase 2	https://ClinicalTrials.gov/show/NCT03691909
22	NCT04355728	Use of UC-MSCs for COVID-19 Patients	Completed	Corona Virus Infection|ARDS|ARDS, Human|Acute Respiratory Distress Syndrome|COVID-19	Phase 1|Phase 2	https://ClinicalTrials.gov/show/NCT04355728
23	NCT01087996	The Percutaneous Stem Cell Injection Delivery Effects on Neomyogenesis Pilot Study (The POSEIDON-Pilot Study)	Completed	Stem Cell Transplantation	Phase 1|Phase 2	https://ClinicalTrials.gov/show/NCT01087996
24	NCT03059355	Infusion of Umbilical Cord Versus Bone Marrow Derived Mesenchymal Stem Cells to Evaluate Cytokine Suppression.	Terminated	Endothelial Dysfunction|Metabolic Syndrome|Chronic Inflammation	Phase 1|Phase 2	https://ClinicalTrials.gov/show/NCT03059355
25	NCT03925324	Serial Infusions of Allogeneic Mesenchymal Stem Cells in Cardiomyopathy Patients with Left Ventricular Assist Device	Terminated	Ischemic Heart Disease|Non-ischemic Cardiomyopathy	Phase 2	https://ClinicalTrials.gov/show/NCT03925324
26	NCT03799718	Safety and Efficacy of Repeated Administration of NurOwn (MSC-NTF Cells) in Participants with Progressive MS	Completed	Multiple Sclerosis, Chronic Progressive	Phase 2	https://ClinicalTrials.gov/show/NCT03799718
27	NCT02958267	Investigation of Mesenchymal Stem Cell Therapy for the Treatment of Osteoarthritis of the Knee	Completed	Knee Osteoarthritis	Phase 2	https://ClinicalTrials.gov/show/NCT02958267
28	NCT03857841	A Safety Study of IV Stem Cell-derived Extracellular Vesicles (UNEX-42) in Preterm Neonates at High Risk for BPD	Terminated	Bronchopulmonary Dysplasia	Phase 1	https://ClinicalTrials.gov/show/NCT03857841
29	NCT01909154	Safety Study of Local Administration of Autologous Bone Marrow Stromal Cells in Chronic Paraplegia	Completed	Spinal Cord Injury	Phase 1	https://ClinicalTrials.gov/show/NCT01909154
30	NCT01733186	Evaluation of Safety and Exploratory Efficacy of CARTISTEMÂ^®^, a Cell Therapy Product for Articular Cartilage Defects	Completed	Degeneration Articular Cartilage Knee	Phase 1|Phase 2	https://ClinicalTrials.gov/show/NCT01733186
31	NCT01392625	PercutaneOus StEm Cell Injection Delivery Effects on Neomyogenesis in Dilated CardioMyopathy (The POSEIDON-DCM Study)	Completed	Non-ischemic Dilated Cardiomyopathy	Phase 1|Phase 2	https://ClinicalTrials.gov/show/NCT01392625
32	NCT03117738	A Study to Evaluate the Safety and Efficacy of AstroStem in Treatment of Alzheimer’s Disease	Completed	Alzheimer Disease	Phase 1|Phase 2	https://ClinicalTrials.gov/show/NCT03117738
33	NCT02674399	A Phase 2 Study to Evaluate the Efficacy and Safety of JointStem in Treatment of Osteoarthritis	Completed	Osteoarthritis, Knee	Phase 2	https://ClinicalTrials.gov/show/NCT02674399
34	NCT04348435	A Randomized, Double-Blind, Single Center, Efficacy and Safety Study of Allogeneic HB-adMSCs Against COVID-19.	Completed	COVID-19	Phase 2	https://ClinicalTrials.gov/show/NCT04348435
35	NCT00768066	The Transendocardial Autologous Cells (hMSC or hBMC) in Ischemic Heart Failure Trial (TAC-HFT)	Completed	Stem Cell Transplantation|Ventricular Dysfunction, Left	Phase 1|Phase 2	https://ClinicalTrials.gov/show/NCT00768066
36	NCT02467387	A Study to Assess the Effect of Intravenous Dose of (aMBMC) to Subjects with Non-ischemic Heart Failure	Completed	Non-Ischemic Heart Failure	Phase 2	https://ClinicalTrials.gov/show/NCT02467387
37	NCT00629018	Safety and Efficacy Study of Stem Cell Transplantation to Treat Dilated Cardiomyopathy	Completed	Dilated Cardiomyopathy	Phase 2	https://ClinicalTrials.gov/show/NCT00629018
38	NCT04491240	Evaluation of Safety and Efficiency of Method of Exosome Inhalation in SARS-CoV-2 Associated Pneumonia.	Completed	Covid19|SARS-CoV-2 PNEUMONIA|COVID-19	Phase 1|Phase 2	https://ClinicalTrials.gov/show/NCT04491240
39	NCT01152580	Melatonin Osteoporosis Prevention Study	Completed	Osteoporosis|Osteopenia	Phase 1	https://ClinicalTrials.gov/show/NCT01152580
40	NCT03060551	Injection of Autologous Adipose-derived Stromal Vascular Fraction in the Finger of Systemic Sclerosis Patients	Completed	Systemic Sclerosis	Early Phase 1	https://ClinicalTrials.gov/show/NCT03060551
41	NCT02886884	Allogeneic Mesenchymal Human Stem Cells Infusion Therapy for Endothelial DySfunctiOn in Diabetic Subjects	Completed	Diabetes Mellitus, Type 2	Phase 1|Phase 2	https://ClinicalTrials.gov/show/NCT02886884
42	NCT01529008	Study on Autologous Osteoblastic Cells Implantation to Early-Stage Osteonecrosis of the Femoral Head	Terminated	Osteonecrosis of the Femoral Head	Phase 3	https://ClinicalTrials.gov/show/NCT01529008
43	NCT00927355	Effect of Thiazolidinediones on Human Bone	Completed	Osteoblast|Adipocytes|Bone Density|Osteocalcin|Adiponectin|Mesenchymal Stem Cells	Not Applicable	https://ClinicalTrials.gov/show/NCT00927355
44	NCT02165904	Subarachnoid Administrations of Adults Autologous Mesenchymal Stromal Cells in SCI	Completed	Spinal Cord Injury	Phase 1	https://ClinicalTrials.gov/show/NCT02165904
45	NCT02859415	Continuous 24 h Intravenous Infusion of Mithramycin, an Inhibitor of Cancer Stem Cell Signalling, in People with Primary Thoracic Malignancies or Carcinomas, Sarcomas or Germ Cell Neoplasms with Pleuropulmonary Metastases	Terminated	Esophageal Neoplasms|Lung Neoplasms|Mesothelioma|Thymus Neoplasms|Neoplasms, Germ Cell and Embryonal	Phase 1|Phase 2	https://ClinicalTrials.gov/show/NCT02859415
46	NCT01270139	Plasmonic Nanophotothermal Therapy of Atherosclerosis	Completed	Stable Angina|Heart Failure|Atherosclerosis|Multivessel Coronary Artery Disease	Not Applicable	https://ClinicalTrials.gov/show/NCT01270139
47	NCT01771913	Immunophenotyping of Fresh Stromal Vascular Fraction From Adipose Derived Stem Cells (ADSC) Enriched Fat Grafts	Completed	Breast Reconstruction|Contour Irregularities|Volume Insufficiency	Phase 2	https://ClinicalTrials.gov/show/NCT01771913
48	NCT02037204	IMPACT: Safety and Feasibility of a Single-stage Procedure for Focal Cartilage Lesions of the Knee.	Completed	Foreign-Body Reaction|Inflammation|Effusion (L) Knee|Knee Pain Swelling	Phase 1|Phase 2	https://ClinicalTrials.gov/show/NCT02037204
49	NCT00957931	Allo-HCT MUD for Non-malignant Red Blood Cell (RBC) Disorders: Sickle Cell, Thal, and DBA: Reduced Intensity Conditioning, Co-tx MSCs	Completed	Sickle Cell Disease|Thalassemia|Diamond-Blackfan Anemia	Phase 2	https://ClinicalTrials.gov/show/NCT00957931
50	NCT02336230	A Prospective Study of Remestemcel-L, Ex-vivo Cultured Adult Human Mesenchymal Stromal Cells, for the Treatment of Pediatric Participants Who Have Failed to Respond to Steroid Treatment for Acute Graft-Versus-Host Disease (aGVHD)	Completed	Grade B aGVHD|Grade C aGVHD|Grade D aGVHD	Phase 3	https://ClinicalTrials.gov/show/NCT02336230
51	NCT01460901	Study of Donor Derived, Multi-virus-specific, Cytotoxic T-Lymphocytes for Relapsed/Refractory Neuroblastoma	Completed	Neuroblastoma	Phase 1	https://ClinicalTrials.gov/show/NCT01460901
52	NCT00927784	Effect of Intramyocardial Injection of Mesenchymal Precursor Cells on Heart Function in People Receiving an LVAD	Terminated	Heart Failure	Phase 2	https://ClinicalTrials.gov/show/NCT00927784
53	NCT01781390	Safety Study of Allogeneic Mesenchymal Precursor Cell Infusion in Myocardial Infarction	Completed	Acute Myocardial Infarction	Phase 2	https://ClinicalTrials.gov/show/NCT01781390
54	NCT01861054	Pilot Study to Evaluate Safety and Biological Effects of Orally Administered Reparixin in Early Breast Cancer	Terminated	Breast Cancer	Phase 2	https://ClinicalTrials.gov/show/NCT01861054
55	NCT02001974	Pilot Study to Evaluate Reparixin With Weekly Paclitaxel in Patients With HER 2 Negative Metastatic Breast Cancer (MBC)	Completed	Metastatic Breast Cancer	Phase 1	https://ClinicalTrials.gov/show/NCT02001974
56	NCT03473301	A Study of UCB and MSCs in Children With CP: ACCeNT-CP	Completed	Cerebral Palsy	Phase 1|Phase 2	https://ClinicalTrials.gov/show/NCT03473301

**Table 2:** The role of miRNA in differentiation of MSCs.

MSC Differentiation	miRNAs	Target Genes/Pathways	Promotion/Inhibition	References
**Osteogenesis**	miR-133/miR-135	RUNX2/SMAD5	Inhibition	[109]
	miR-133a-3p	MEG3	Inhibition	[110]
	miR-138	ALP, RUNX2	Inhibition	[111]
	miR-138	FAK, ERK1/2, RUNX2	Inhibition	[112]
	miR-125b	ErbB2	Inhibition	[113]
	miR-27a/miR-489	GCA/PEX7/APL	Inhibition	[114]
	miR-27a	Sp7	Inhibition	[115]
	miR-204/211	RUNX2	Inhibition	[116]
	miR-206	Cx43	Inhibition	[117]
	miR-26a	SMAD1	Inhibition	[118]
	miR-200a-3p	Glutaminase	Inhibition	[119]
	miR-185	Bgn, BMP/SMAD	Inhibition	[120]
	miR-125a-3p	SMAD4 and JAK1	Inhibition	[121]
	miR-141/miR-200a	SVCT2	Inhibition	[122]
	miR-384-5p	Gli2	Inhibition	[123]
	miR-23a	BMPR1B, CXCL12	Inhibition	[124,125]
	miR-23a	LRP5	Inhibition	[126]
	miR-23a-5p	MAPK13	Inhibition	[127]
	miR-23b	RUNX2	Inhibition	[128]
	miR-378	Wnt/β-catenin signaling	Inhibition	[129]
	miR-186	SIRT6	Inhibition	[130]
	let-7a-5p	TGFβR1	Inhibition	[131]
	miR-9-5p	Wnt3a	Inhibition	[132]
	miR-10	RUNX2	Inhibition	[133]
	miR-16-2-3p	Wnt5a	Inhibition	[134]
	miR-17	Smurf1	Inhibition	[135]
	miR-17-5p/miR-106a	BMP2	Inhibition	[135]
	miR-24	TCF-1	Inhibition	[136]
	miR-30	RUNX2, SMAD1	Inhibition	[137]
	miR-31	SATB2 and OSX	Inhibition	[138,139,140]
	miR-145	CBFB	Inhibition	[141]
	miR-93-5p	BMP2	Inhibition	[142]
	miR-96	Osterix	Inhibition	[143]
	miR-98	BMP2	Inhibition	[144]
	miR-100	BMPR2	Inhibition	[145]
	miR-124	Sp7	Inhibition	[146]
	miR-125b	BMPR1B	Inhibition	[147]
	miR-132	β-catenin	Inhibition	[148]
	miR-135b	IBSP and OSX	Inhibition	[149]
	miR-137	RUNX2	Inhibition	[150]
	miR-139-5p	β-catenin, FZD4	Inhibition	[151]
	miR-140-5p	BMP2	Inhibition	[152]
	miR-143	RUNX2	Inhibition	[128]
	miR-144-3p	SMAD4	Inhibition	[153]
	miR-153	BMPR2	Inhibition	[154]
	miR-154-5p	Wnt11	Inhibition	[155]
	miR-183-5p	Hmox1	Inhibition	[156]
	miR-195-5p	BMPR1A	Inhibition	[157]
	miR-203	RUNX2	Inhibition	[128]
	miR-203-3p	SMAD1	Inhibition	[158]
	miR-204	RUNX2, BMP2	Inhibition	[116,159]
	miR-205	RUNX2, SATB2	Inhibition	[160]
	miR-214	BMP2	Inhibition	[161]
	miR-214-5p	COL4A1	Inhibition	[162]
	miR-217	RUNX2	Inhibition	[163]
	miR-221	RUNX2	Inhibition	[128]
	miR-221-5p	SMAD3	Inhibition	[164]
	miR-222-3p	RUNX2, SMAD5	Inhibition	[165]
	miR-335	RUNX2	Inhibition	[166]
	miR-338-3p	RUNX2, FGFR2	Inhibition	[167]
	miR-381	Wnt5a, FZD3	Inhibition	[168]
	miR-383	SATB2	Inhibition	[169]
	miR-433	RUNX2	Inhibition	[170]
	miR-486-5p	SIRT1	Inhibition	[171]
	miR-503-5p	RUNX2	Inhibition	[172]
	miR-708	SMAD3	Inhibition	[173]
	miR-1297	Wnt5a	Inhibition	[174]
	miR-376c-3p	IGF1R/Akt	Inhibition	[175]
	miR-1305	RUNX2	Inhibition	[176]
	miR-146a	SMAD4	Inhibition	[177]
	miR-637	SP7	Inhibition	[178]
	miR-29a	HDAC4	Promotion	[179]
	miR-196a	HOXC8	Promotion	[180]
	miR-7-5p	CMKLR1	Promotion	[181]
	miR-224	Rac1	Promotion	[182]
	miR-210	ACVR1b	Promotion	[183]
	miR-2861	HDAC5	Promotion	[184]
	miR-148b	Unknown	Promotion	[114]
	miR-217	DKK1	Promotion	[185]
	let-7/miR-24/miR-125b/miR-138	Unknown	Promotion	[186]
	miR-200c	Myd88, AKT/β-catenin	Promotion	[187]
	miR-21	PTEN, PI3K/Akt/HIF-1α	Promotion	[188]
	miR-9	DKK1	Promotion	[189]
	miR-10b	SMAD2	Promotion	[190]
	miR-17-5p	SMAD7	Promotion	[191]
	miR-21-5p	SMAD7	Promotion	[192]
	miR-26b	GSK3β	Promotion	[193]
	miR-34a	NOTCH2 and HES1	Promotion	[194]
	miR-378	None validated	Promotion	[195]
	miR-346	GSK-3β	Promotion	[196]
	miR-10a	KLF4	Promotion	[197]
	miR-322	Tob2	Promotion	[198]
	miR-21	Spry1	Promotion	[199]
	miR-96	SOX9, aggrecan and FABP4	Promotion	[200]
	miR-22	HDAC6	Promotion	[201]
	miR-218	SFRP2 and DKK2	Promotion	[202]
	miR-199b-5p	GSK3β	Promotion	[203]
	miR-335-5p	DKK1	Promotion	[204]
	miR-433-3p	DKK1	Promotion	[205]
	miR-590-3p	APC	Promotion	[206]
	miR-27a	PPARγ, GREM1	Promotion	[207]
	miR-26a	Runx2, OC, GSK3β	Promotion	[208,209]
	miR-148a	IGF1	Promotion	[210]
	miR-200b	Cx43, VEGF-A	Promotion	[211]
	miR-92a	SMAD6	Promotion	[212]
	miR-9	RUNX2, ERK	Promotion	[151]
	miR-590-5p	SMAD7	Promotion	[213]
	miR-130a-3p	SIRT7	Promotion	[214]
	miR-497-5p	Smurf2	Promotion	[215]
**Chondrogenesis**	miR-199a	SMAD1	Inhibition	[216]
	miR-29a	FOXO3A	Inhibition	[217]
	miR-124	NFATC1	Inhibition	[218]
	miR-182-5p	PTHLH	Inhibition	[219]
	miR-30a	SOX9	Inhibition	[220]
	miR-30b	SOX9	Inhibition	[221]
	miR-145/miR-495	SOX9	Inhibition	[222,223]
	miR-449a	LEF-1	Inhibition	[224]
	miR-574-3p	RXRα	Inhibition	[225]
	miR-221	TRPS1/MDM2	Inhibition	[226]
	miR-483	SMAD4	Inhibition	[227]
	miR-143-3p/miR-125b	BMPR2	Inhibition	[228,229]
	miR-26b	Wnt	Inhibition	[230]
	miR-23c	FGF2	Inhibition	[231]
	miR-29b	HDAC4	Inhibition	[232]
	miR-194	SOX5	Inhibition	[233]
	miR-193b	TGFB2 and TGFBR3	Inhibition	[234]
	miR-140	SOX9/COL2A1/HDAC4	Promotion	[235,236]
	miR-140-5p	RALA/FZD6/GALNTL1,Wnt	Promotion	[237,238]
	miR-335-5p	Daam1/ROCK1/DKK1,Wnt/β-catenin/TCF	Promotion	[239]
	miR-30a	DLL4, Notch	Promotion	[240]
	miR-95-5p	HDAC2/8	Promotion	[241]
	miR-193b-3p	HDAC3	Promotion	[242]
	miR-320c	CDK6	Promotion	[243]
	miR-526b-3p/miR-590-5p	SAMD7	Promotion	[244]
	miR-132-3p	ADAMTS-5	Promotion	[245]
	miR-149-5p	FUT-1	Promotion	[246]
	miR-892b	KLF10, TGF-β/SMAD and Ihh	Promotion	[247]
	miR-520d-5p	HDAC1	Promotion	[248]
	miR-127-5p	SOX9/RUNX2	Promotion	[249]
	miR-638/miR-663	Unknown	Unknown	[250]
**Adipogenesis**	miR-138	EID-1	Inhibition	[251]
	miR-31	CEBPA	Inhibition	[252]
	miR-363	E2F3	Inhibition	[253]
	miR-540	PPARγ	Inhibition	[254]
	miR-301b/miR-130b	PPARγ	Inhibition	[255]
	miR-330-5p	RXRγ	Inhibition	[256]
	miR-27b	LPL	Inhibition	[257]
	miR-377-3p	LIFR	Inhibition	[258]
	miR-31-5p	C/EBP-α	Inhibition	[259]
	miR-431	IRS2	Inhibition	[260]
	miR-27b	PPARg and C/EBPα	Inhibition	[261]
	miR-155/miR-221/miR-222	CEBPB, CDKN1B, PIK3R1	Inhibition	[262]
	miR-143	MAP2K5	Promotion	[263]
	miR-26a	PTEN, Cyclin E1, CDK6	Promotion	[264]
	miR-30a-5p	C8orf4	Promotion	[265]
	miR-199a-3p	KDM6A/WNT	Promotion	[266]
	miR-320	RUNX2	Promotion	[267]
	hsa-mir 199a/hsa-mir346	LIF	Promotion	[268]
	miR-642a-3p	Unknown	Promotion	[269]
	miR-30a and 30d	RUNX2	Promotion	[269]
	miR-21	TGFBR2	Promotion	[270]
	miR-26	ADAM17	Promotion	[271]
	miR-30c	PAI-1 and ALK2	Promotion	[272]
**Myogenesis**	miR-124	Dlx5	Inhibition	[273]
	miR-124-3p	Cav1	Promotion	[274]
	miR-139-5p	Wnt/β-catenin	Promotion	[151]
**Neurogenesis**	miR-218	Wnt	Promotion	[275]
	miR-142-5p	RhoA/ROCK1	Promotion	[276]
	miR-130a/miR-206	TAC1	Promotion	[277]

**Table 3:** The role of lncRNA in differentiation of MSCs.

MSC Differentiation	LncRNAs	Target Genes/Pathways	Promotion/Inhibition	References
**Osteogenesis**	H19	miR-141, miR-22/Wnt/β-catenin	Promotion	[278]
	H19	miR-675/TGF-β1/SMAD3/HDAC	Promotion	[279]
	H19	miR-138/FAK	Promotion	[280]
	MEG3	SOX2/BMP4	Promotion	[281]
	MEG3	miR-133a-3p	Inhibition	[110]
	MEG3	EZH2/Wnt	Inhibition	[282]
	MEG3	miR-140-5p	Promotion	[283]
	DANCR	p38 MAPK pathway	Inhibition	[284]
	MALAT1	miR-34c/SATB2	Promotion	[285]
	MALAT1	miR-143/OSX	Promotion	[286]
	HULC	miR-195	Promotion	[287]
	PGC1β-OT1	miR-148a-3p/KDM6B	Promotion	[288]
	OG	hnRNPK/BMP	Promotion	[289]
	AK141205	CXCL13	Promotion	[290]
	NONHSAT009968	-	Inhibition	[291]
	TCONS_00041960	miR-204-5p and miR-125a-3p	Promotion	[292]
	AK028326	CXCL13	Promotion	[293]
**Chondrogenesis**	DANCR	miR-1305/SMAD4 axis	Promotion	[294]
	LOC102723505 (ROCR)	SOX9	Promotion	[40]
	ZBED3-AS1	zbed3 and Wnt/β-catenin	Promotion	[295]
**Adipogenesis**	H19	CTCF/H19/miR-675/HDAC	Inhibition	[296]
	MEG3	miR-140-5p	Inhibition	[283]
	PGC1β-OT1	miR-148a-3p/KDM6B	Inhibition	[288]
	ROA	hnRNP A1/PTX3/ERK	Inhibition	[297]
	lnc13728	ZBED3/Wnt/β-catenin	Promotion	[298]
	GAS5	miR-18a/CTGF axis	Inhibition	[299]
	HOTAIR	-	Inhibition	[300]
	TCONS_00041960	miR-204-5p and miR-125a-3p	Inhibition	[292]
**Myogenesis**	HULC	BMP9/Wnt/β-catenin/Notch	Promotion	[301]
**Neurogenesis**	H19	miR-675/IGFR	Inhibition	[302]

**Table 4:** List of lncRNA–miRNA axes regulating differentiation of MSCs into distinct cell lineages.

Progenitor	Differentiation		lncRNA		miRNA		mRNA		Ref.
	Type	Rate	Type	Level	Type	Level	Type	Level	
BMMSC	Angiogenic	↑	MALAT1	↑	miR-206	↓	VEGFA	↑	[305]
AMSC	Adipogenic	↑	H19	↑	miR-30a	↓	C8ORF4	↑	[265]
BMMSC	Adipogenic	↑	SNHG15	↑	miR-497	↓	RUNX2	↑	[311]
BMMSC	Adipogenic	↑	LOXL1-AS1	↑	miR-196a-5p	↓	HMGA2	↑	[312]
AMSC	Adipogenic	↓	HCG11	↑	miR-204-5p	↓	SIRT1	↑	[313]
BMMSC	Adipogenic	↓	TCONS_00023297	↑	miR-608	↓	RUNX	↑	[314]
BMMSC	Adipogenic	↓	GAS5	↑	miR-365	↓	–	–	[315]
hMSC	Chondrogenic	↑	ADAMTS9-AS2	↑	miR-942-5p	↓	SCRG1	↑	[316]
UCMSC	Chondrogenic	↑	H19	↑	miR-29b-3p	↓	SOX9	↑	[317]
PBMSC	Chondrogenic	↓	DNM3OS	↑	miR-127-5p	↓	GREM2	↑	[318]
SMSC	Chondrogenic	↓	XIST	↑	miR-27b-3p	↓	ADAMTS-5	↑	[319]
BMMSC	Hepatogenic	↑	MALAT1	↑	miR-217	↓	ZEB1	↑	[320]
BMMSC	Hepatogenic	↓	SNHG1	↑	miR-15a	↓	SMURF1	↑	[321]
ADMSC	Lymphatogenic	↑	MIAT	↑	miR-495	↓	PROX1	↑	[322]
BMMSC	Osteogenic	↑	HOTAIRM1	↑	miR-152-3p	↓	ETS1	↑	[323]
BMMSC	Osteogenic	↑	TUG	↑	miR-204	↓	SIRT1	↑	[324]
BMMSC	Osteogenic	↑	MALAT1	↑	miR-96	↓	OSX	↑	[308]
BMMSC	Osteogenic	↑	MALAT1	↑	miR-129-5p	↓	–	–	[309]
BMMSC	Osteogenic	↑	SNHG5	↑	miR-212-3p	↓	GDF5	↑	[325]
BMMSC	Osteogenic	↑	KCNQ1OT1	↑	miR-29b-3p	↓	–	–	[326]
BMMSC	Osteogenic	↑	FGD5-AS1	↑	miR-506-3p	↓	BMP7	↑	[327]
hMSC	Osteogenic	↑	LINC00657	↑	miR-214-3p	↓	BMP2	↑	[328]
ASMSC	Osteogenic	↓	MEG3	↑	miR-125a-5p	↓	TNFAIP3	↑	[329]
BMMSC	Osteogenic	↓	LINC01234	↑	miR-513a-5p	↓	AOX1	↑	[330]
BMMSC	Osteogenic	↓	MIAT	↑	miR-150-5p	↓	–	–	[331]
BMMSC	Osteogenic	↓	MIR99AHG	↑	miR-4660	↓	OSX	↑	[332]
BMMSC	Osteogenic	↓	HOTAIR	↑	miR-378g	↓	NNMT	↑	[333]
UCMSC	Osteogenic	↓	LINC02381	↑	miR-21	↓	KLF12	↑	[334]

AMSC: adipose-derived MSC; ASMSC: ankylosing spondylitis patient-derived MSC; BMMSC: bone marrow-derived MSC; hMSC: human MSC; PBMSC: peripheral blood-derived MSC; SMSC: synovium-derived MSC; UCMSC: umbilical cord-derived MSC.
